# Molecular Identification of Biofilm-producing *Staphylococcus aureus* in Contact Lens Storage Solutions

**DOI:** 10.18502/jovr.v21.13795

**Published:** 2026-09-23

**Authors:** Mehrnaz Atighehchian, Manoochehr Shabani, Zohreh Abedinifar, Zahra Manoochehri, Mehran Zarei-Ghanavati

**Affiliations:** ^1^Jahrom University of Medical Sciences, Jahrom, Iran; ^2^Noor Ophthalmology Research Center, Noor Eye Hospital, Tehran, Iran; ^3^Zoonoses Research Center, Jahrom University of Medical Sciences, Jahrom, Iran; ^4^Microbiology Unit, Department of Pathology, Farabi Eye Hospital, Tehran University of Medical Sciences, Tehran, Iran; ^5^Farabi Eye Hospital, Tehran University of Medical Sciences, Tehran, Iran

**Keywords:** Biofilm, Contact Lens Storage Solution, ica Genes, Keratitis, Staphylococcus aureus

## Abstract

**Purpose:**

This study aimed to evaluate the presence of biofilm-producing *Staphylococcus aureus* in contact lens storage solutions (CLSS) used for both cosmetic and vision-correcting purposes. Microbiological assays and molecular methods were used to detect biofilm-associated genes, specifically *icaA* and *icaD*. The findings could provide molecular evidence of contamination in CLSS and identify behavioral risk factors associated with biofilm-forming strains.

**Methods:**

A total of 162 CLSS samples were collected from asymptomatic students living in dormitories and hospital staff. Bacterial isolation and identification were performed using standard culture methods and biochemical tests. Biofilm formation was evaluated phenotypically, and molecular confirmation of *S. aureus* and biofilm-associated genes (*icaA* and *icaD*) was performed using polymerase chain reaction (PCR). Associations between contamination and lens type, and also user behaviors, including lens-washing practices, showering, and swimming while wearing lenses, were analyzed.

**Results:**

This study evaluated 162 CLSS samples from 162 asymptomatic participants (mean age: 24.8 ± 4.2 years). Of these, 148 subjects (91.35%) were females. *Staphylococcus aureus* contamination was detected in7 of 162 samples (4.32%),and 4 of the 7 isolates (57.14%) carried biofilm-associated *icaA* and *icaD* genes. *Staphylococcus aureus* contamination was found in 5 of 108 CLSS used for vision-correcting purposes (4.62%; *P* = 0.53). Contamination was significantly associated with lack of lens washing before wearing, which occurred in 4 of 141 participants (2.83%; *P* = 0.005), and lack of hand washing before handling contact lenses, which was observed in 7 of 159 participants (4.40%; *P* = 0.043).

**Conclusion:**

This study provides molecular evidence that CLSS can act as reservoirs for biofilm-producing *S. aureus*. The detection of *icaA*- and *icaD*-positive strains highlights the risk of persistent contamination and the potential development of contact lens-related infectious keratitis. Modifiable behaviors, especially proper lens washing, may significantly reduce the risk of contamination with biofilm-producing *S. aureus.*

##  INTRODUCTION 

*Staphylococcus aureus* (*S. aureus*) is a common pathogen responsible for various local and systemic infections. It can be found on the skin, hands, nasopharynx, and mucous membranes.^[1,2,3]^ This pathogen has many virulence factors, including extracellular toxins, fibronectin, fibrinogen, laminin, and elastin.^[1]^ These virulence factors facilitate tissue colonization and help bacteria evade the host immune system.^[12]^ Additionally, this bacterium can form a structure called a biofilm, especially when it adheres to medical devices.^[4,5]^ Biofilms are multicellular communities embedded in a self-produced extracellular matrix composed of proteins, polysaccharides, and other substances.^[4,5][6][7][8][9]^ Detection of virulence-associated genes is important for identifying biofilm-forming microorganisms. Several biofilm-associated genes have been identified, including the intercellular adhesion genes (*icaA* and *icaD*).^[10,11,13]^ Approximately 65% of hospital-acquired infections, such as those caused by methicillin-resistant *Staphylococcus aureus *(MRSA), are linked to biofilm formation.^[6,7,8,9,10]^

*Staphylococcus aureus* is a common cause of serious ocular infections, such as contact lens-related infectious keratitis, and the biofilm produced b*y* thisorganism can contribute to persistent infections and resistance to treatment.^[12,13,14,15]^ While several studies have reported biofilm-forming *S. aureus* in cases of contact lens-related infectious keratitis, there is limited molecular evidence concerning biofilm-producing strains in contact lens storage solutions (CLSS). This study aimed to isolate and identify biofilm-forming *S. aureus* from CLSS through both microbiological and molecular methods. The findings suggest that CLSS may act as potential reservoirs for this organism.

##  METHODS

This cross-sectional study was conducted at Jahrom University of Medical Sciences, Jahrom, Iran. The study adhered to the tenets of the Declaration of Helsinki, and the study protocol was approved by the Ethics Committee of Jahrom University of Medical Sciences (approval No. IR.JUMS.REC.13970104). Written informed consent was obtained from all participants for the use of their CLSS in research.

A total of 162 CLSS samples were collected from 162 asymptomatic students living in dormitories and hospital staff. The sample size was determined using the Cochran formula. Inclusion criteria were participants aged 18 to 45 years who had used contact lenses for cosmetic or vision-correcting purposes for at least 1 year, wore their lenses for up to 16 hours per day, and were willing to participate in the study. Exclusion criteria included individuals who wore contact lenses for more than 16 hours per day or overnight, and individuals with ocular surface diseases, systemic disorders (e.g., diabetes mellitus), or a history of ocular surgery.

Participants were evaluated for proper hand hygiene before handling contact lenses, cleaning the lenses with multipurpose solutions before use, showering while wearing lenses, and for the type of lenses used.

### Sample Preparation

After shaking each CLSS sample from both sides of the storage for 1 minute, 100 µL of the fluid was transferred to a 1.5 mL Eppendorf tube containing brain heart infusion (BHI) medium and incubated at 37ºC for 24 hours prior to analysis.

### Bacterial Identification

All CLSS samples were evaluated using Gram staining to confirm the presence of * S. aureus* Gram-positive cocci. For bacterial isolation, samples from the BHI medium were cultured on blood agar, Eosin Methylene Blue (EMB) agar, and mannitol salt agar. To confirm *S. aureus* growth, several biochemical tests were performed, including catalase and oxidase tests.

### Bacterial DNA Extraction

Genomic DNA was extracted using a DNA extraction kit (Cinnagen, Tehran, Iran) according to the manufacturer's instructions.

### Polymerase Chain Reaction (PCR)

Polymerase Chain Reaction (PCR) amplification was carried out using a thermocycler (Eppendorf, Germany). The cycling conditions, including specific annealing temperatures and cycle times, were optimized for the *S. aureus* genome primers. A 20 µL PCR reaction mixture was prepared using a commercial PCR premix (Bioneer, Iran) according to the manufacturer's protocol and the primer conditions listed in Table 1.

### Identification of Molecular S. aureus Biofilm

Bacterial isolates obtained from culture media were confirmed as *S. aureus* using PCR amplification. The presence of biofilm-associated genes (*icaA* and *icaD*) was detected using specific primers under the PCR conditions described in Tables 2 and 3.

### DNA Electrophoresis

PCR products were visualized by electrophoresis on a 1.5% agarose gel stained with ethidium bromide and photographed under UV illumination.

### Statistical Analysis

All data were analyzed using SPSS software (version 27, IBM Corp., Armonk, NY, USA). The normality of the data was assessed using the Shapiro–Wilk test. The chi-square test was used to determine the relationships between variables. A *P*-value of < 0.05 was considered statistically significant.

##  RESULTS

A total of 162 CLSS samples were collected from 162 asymptomatic participants (148 [91.35%] female; mean age: 24.8 ± 4.2 years). Among these, seven samples (4.32%) were found to be contaminated with *S. aureus*, and all contaminated samples were collected from female participants. All seven contaminated CLSS were confirmed using *S. aureus*-specific primers (nuc) [Figures 1 & 2]. Four of the seven isolates (57.14%) carried both the *icaA* and *icaD* genes, which are known for biofilm production. Five (71.42%) contaminated samples were obtained from CLSS used for vision correction. There was no significant association between the type of contact lens and the growth of* S. aureus* (*P* = 0.53) [Table 4].

**Table 1:** PCR reaction components and conditions

**Component**	**Concentration**	**Volume (µL)**	**Final concentration**	**Description**
PCR PreMix	—	—	—	Contains Tris-HCl, MgCl₂, dNTPs, and Taq DNA Polymerase
Tris-HCl	10 mM	—	—	Buffer component
MgCl₂	1.5 mM	—	—	Cofactor for DNA polymerase
KCl	30 mM	—	—	Ionic strength stabilizer
dNTPs	250 µM	—	—	Nucleotide mix
Taq DNA polymerase	1 U	—	—	Thermostable DNA polymerase
Forward primer	—	1	5-10 pmol	Specific primer sequence
Reverse primer	—	1	5-10 pmol	Specific primer sequence
DNA template	—	5	5-50 ng	Target DNA
Distilled water	—	13	—	Brings total volume to 20 µL
Total volume	—	20	—	—
PCR, polymerase chain reaction.				

**Table 2:** Primers used for the detection of *Staphylococcus aureus*

**Bacterium**	**Gene**	**Primer name**	**Sequence (5' → 3')**	**Product size (bp)**
*Staphylococcus aureus*	nuc	NucF	GCGATTGATGGTGATACGGTT	160
*Staphylococcus aureus*	nuc	NucR	AGCCAAGCCTTGACGAACTAAAGC	—

**Table 3:** Primers used for the detection of biofilm-associated genes in *Staphylococcus aureus*

**Bacterium**	**Gene**	**Primer name**	**Sequence (5' → 3')**	**Product size (bp)**
*Staphylococcus aureus*	*icaA*	Forward	ACACTTGCTGGCGCAGTCAA	188
*Staphylococcus aureus*	*icaA*	Reverse	TCTGGAACCAACATCCAACA	—
*Staphylococcus aureus*	*icaD*	Forward	ATGGTCAAGCCCAGACAGAG	198
*Staphylococcus aureus*	*icaD*	Reverse	AGTATTAATGTTTAAAGCAA	—

Notably, contamination was significantly higher among the foyr participants who did not wash their lenses with a multipurpose solution before wearing them compared to those who did (*P* = 0.005). Additionally, one instance of *S. aureus* biofilm production was detected in a participant who did not wash their lenses before wearing them. Sixteen participants (9.87%) reported showering while wearing contact lenses. Among these, one (6.25%; *P *= 0.29) was found to have a contaminated CLSS. Other demographic and behavioral data are summarized in Table 4.

**Figure 1:**
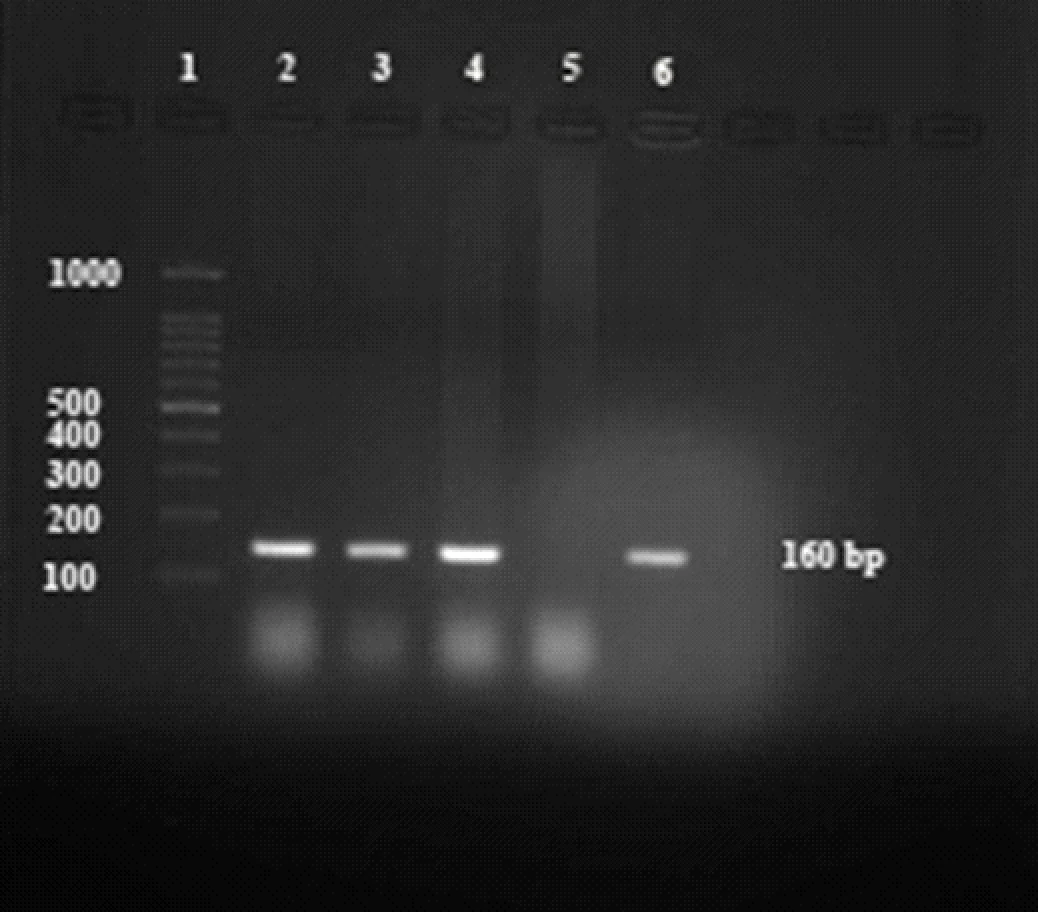
Electrophoresis of PCR product of nuc gene No. 1 = ladder, No. 2-3-4 = isolated *Staphylococcus aureus* samples, No. 5 = negative control, and No. 6 = positive control.

**Figure 2:**
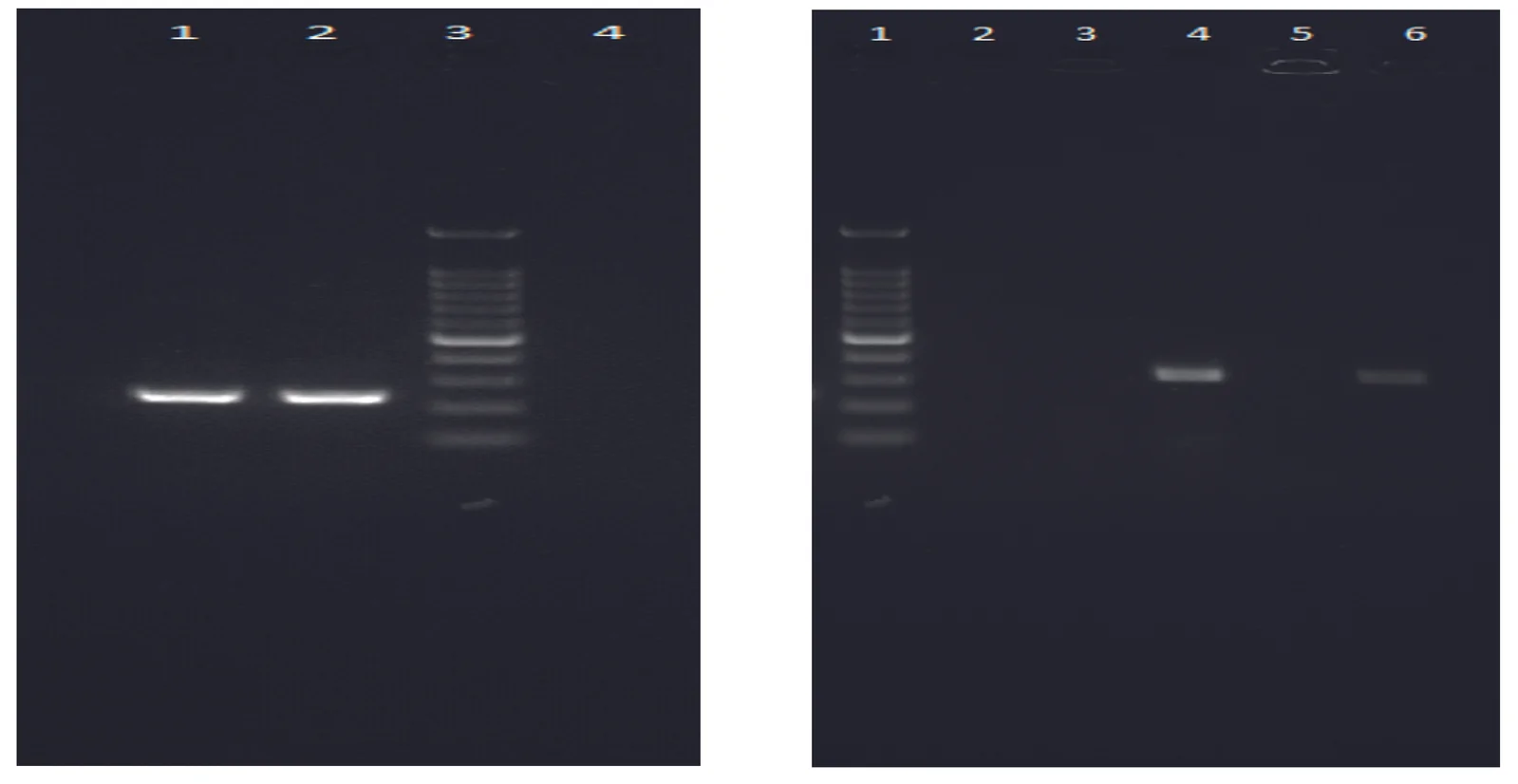
(A) PCR of the *icaA* gene with a band of 188 bp; here, a 50 bp length ladder is used. (B) PCR related to the *icaD* gene with a band of 198 bp; here, a ladder with a length of 50 bp is used.

##  DISCUSSION 

Contact lenses are widely used for vision correction and cosmetic enhancement, with over 140 million users worldwide. Microbial contamination of contact lenses may lead to corneal infection and irreversible ocular complications.^[16,17,18]^

**Table 4:** Demographics and washing habits in asymptomatic participants

**Group**	***Staphylococcus aureus*** ** (%)**	**Biofilm-producing ** ***Staphylococcus aureus *** **(%)**	***P*** **-value**
Vision-correcting lenses	5/108 (4.62%)	4/108 (3.70%)	0.53
Cosmetic contact lenses	2/54 (3.70%)	0	0.53
Female users	7/148 (4.72%)	4/148 (2.70%)	
Students living in dormitories	6/95 (6.31%)	4/95 (4.21%)	
Lack of hand washing before wearing	7/159 (4.40%)	3/159 (1.88%)	0.043
Lack of contact lenses washing	4/141 (2.83%)	1/141 (0.7%)	0.005
Showering with lenses	1/16 (6.25%)	0	0.29
Swimming with lenses	1/1 (100%)	0	0.30
Total	7/162 (4.32%)	4/162 (2.46%)	

*Pseudomonas aeruginosa* is the most common bacterium associated with post-contact lens infectious keratitis. Contamination of contact lenses with Gram-positive bacteria, such *as S. aureus*, can cause peripheral corneal ulceration, and CLSS can be a potential source of infection, leading to severe, sight-threatening complications.^[17,18,19]^ Identifying biofilm-producing bacteria is crucial because biofilm formation may contribute to chronic diseases that are often resistant to standard antimicrobial treatments.^[17,20]^ Previous studies have reported biofilm-forming *S. aureus* in systemic diseases. Mirzaei et al assessed the biofilm formation and the presence of ica genes in *S. aureus* samples collected from the intensive care unit. They demonstrated that biofilm-producing bacteria, which can grow on contaminated intravascular catheters, increased the risk of systemic* S. aureus* infections. Furthermore, microorganisms that produce biofilms tend to be more resistant to antibiotic treatment.^[21]^ Additionally, Khashei et al identified the *icaD* and *icaA* genes and their association with biofilm formation in patients with nosocomial systemic infections caused by *S. aureus*.^[22]^

Most earlier investigations focused on contamination in contact lens storage or on the lenses themselves, while fewer studies specifically examined contamination in CLSS.^[23]^ Evaluating contamination at this level is essential, as it provides crucial insight into potential reservoirs of bacteria that may be overlooked when only cases or lenses are inspected.^[23]^ Additionally, previous studies used conventional microbiological techniques without confirming the presence of specific virulence factors or biofilm genomes. This is significant because organisms that form biofilms tend to be resistant to traditional medications.^[23,5]^ In contrast, the current study employed both standard microbiological diagnostic methods and PCR-based detection of *S. aureus*, including its biofilm-associated genes *icaA* and *icaD*. Our findings showed that four of the seven isolates (57.14%) recovered from CLSS samples contained biofilm-forming *S. aureus*.

Additionally, this study combined microbiological analysis with an assessment of contact lens hygiene practices. While previous research has reported associations between poor hygiene and microbial contamination, few studies have explicitly examined washing behaviors in relation to molecularly confirmed biofilm-forming organisms.^[23,24,25,26]^ Carnt et al reported that water exposure is a common and often underestimated risk behavior among contact lens wearers.^[27]^ Stellwagen et al found that showering while wearing contact lenses significantly increases the risk of microbial keratitis.^[24]^

Our results revealed statistically significant associations between certain behaviors and contamination, including lack of lens washing with multipurpose solutions and lack of hand washing. These findings suggest that simple, modifiable habits can significantly influence CLSS contamination, particularly concerning strains capable of forming biofilms. However, further investigations involving larger cohorts are recommended to confirm these associations and strengthen the evidence regarding the impact of contact lens hygiene practices on CLSS contamination.

It is important to acknowledge some limitations in the present study. The main limitations were the small sample size. Conducting a multicenter study with a larger sample size could minimize sample bias. Additionally, we focused only on *S. aureus* contamination. Future studies should evaluate a broader range of microorganisms, including *Pseudomonas aeruginosa*, *Acanthamoeba*, and various fungal organisms. Furthermore, categorizing participants based on specific contact lens habits and incorporating larger, comparative cohorts will yield more robust and reliable results.

In summary, CLSS can become contaminated with biofilm-forming *S. aureus*, leading to persistent infections and increased resistance to treatment. Therefore, it is crucial to educate contact lens wearers about proper hygiene practices to prevent the serious complications associated with these infections.

##  Financial Support and Sponsorship

None.

##  Conflicts of interest

None.
